# Adapting Mobile and Wearable Technology to Provide Support and Monitoring in Rehabilitation for Dementia: Feasibility Case Series

**DOI:** 10.2196/12346

**Published:** 2019-10-17

**Authors:** Julia Thorpe, Birgitte Hysse Forchhammer, Anja M Maier

**Affiliations:** 1 Engineering Systems Design DTU Management Technical University of Denmark Kongens Lyngby Denmark; 2 Department of Neurology Rigshospitalet, Copenhagen University Hospital Copenhagen Denmark

**Keywords:** dementia, cognitive rehabilitation, mobility, activity, mHealth, uHealth, pervasive health care, P4 health care, health care design

## Abstract

**Background:**

Mobile and wearable devices are increasingly being used to support our everyday lives and track our behavior. Since daily support and behavior tracking are two core components of cognitive rehabilitation, such personal devices could be employed in rehabilitation approaches aimed at improving independence and engagement among people with dementia.

**Objective:**

The aim of this work was to investigate the feasibility of using smartphones and smartwatches to augment rehabilitation by providing adaptable, personalized support and objective, continuous measures of mobility and activity behavior.

**Methods:**

A feasibility study comprising 6 in-depth case studies was carried out among people with early-stage dementia and their caregivers. Participants used a smartphone and smartwatch for 8 weeks for personalized support and followed goals for quality of life. Data were collected from device sensors and logs, mobile self-reports, assessments, weekly phone calls, and interviews. This data were analyzed to evaluate the utility of sensor data generated by devices used by people with dementia in an everyday life context; this was done to compare objective measures with subjective reports of mobility and activity and to examine technology acceptance focusing on usefulness and health efficacy.

**Results:**

Adequate sensor data was generated to reveal behavioral patterns, even for minimal device use. Objective mobility and activity measures reflecting fluctuations in participants’ self-reported behavior, especially when combined, may be advantageous in revealing gradual trends and could provide detailed insights regarding goal attainment ratings. Personalized support benefited all participants to varying degrees by addressing functional, memory, safety, and psychosocial needs. A total of 4 of 6 (67%) participants felt motivated to be active by tracking their step count. One participant described a highly positive impact on mobility, anxiety, mood, and caregiver burden, mainly as a result of navigation support and location-tracking tools.

**Conclusions:**

Smartphones and wearables could provide beneficial and pervasive support and monitoring for rehabilitation among people with dementia. These results substantiate the need for further investigation on a larger scale, especially considering the inevitable presence of mobile and wearable technology in our everyday lives for years to come.

## Introduction

### Background

New approaches are needed to respond to the growing dementia challenge as the population ages [1]. A global action plan recently issued by the World Health Organization calls for solutions to improve the lives of people with dementia and their caregivers and to reduce the impact the condition has on communities [2]. One promising approach is through rehabilitation, defined as a problem-solving process aimed at optimizing social engagement and well-being [3,4]. Rehabilitation among people with cognitive impairment, or cognitive rehabilitation, is characterized by a personalized collaborative approach, which involves setting and working toward individual goals within an everyday life context [5]. Following initial assessment to identify needs and set goals, the rehabilitation process involves two key elements: provision of support to help the person attain their goals and monitoring or evaluation to inform the care strategy for further iterations [4].

Technological advancement over the last decade has led to mobile and wearable technology that could provide both support and monitoring in rehabilitation. The application of personal devices, such as smartphones and wearables, for rehabilitation presents several potential advantages. Regarding support in everyday life, these include the following: wide availability, convenience and familiarity of an existing device, less stigma than specialized assistive technology, and a modular nature that allows for personalization (eg, custom device configurations). Regarding monitoring, sensing capabilities of the latest personal devices offers a scalable and connected approach toward ongoing evaluation. Therefore, this work investigates how smartphones and smartwatches might be applied toward rehabilitation among people with dementia by offering both personalized support in everyday life and objective continuous monitoring of mobility and activity as a means to evaluate function and engagement.

### Mobile and Wearable Technology for Support and Monitoring Among People With Dementia

Various forms of information and communications technology (ICT) have been used for people with dementia to provide functional support in everyday life, improve safety, target psychosocial needs, and support caregivers [6,7]. In the future, a user’s smartphone could provide a single platform through which to offer any combination of similar support features. A host of existing tools for communication, scheduling, reminders, navigation, social, and leisure purposes are already available from off-the-shelf mobile apps. An increasing proportion of elderly people will rely on such tools prior to dementia onset as current users age. Already today, many people with mild cognitive impairment and dementia are using ICT [8], and studies have described interest among members of this population in using wearables for support [9].

Mobile and wearable devices are also packed with sensors that can be used to gather information about users’ lifestyles and behaviors, such as their mobility and activity levels or patterns. Monitoring behavior among people with dementia can provide valuable indicators for functional performance and well-being to inform care strategies [10] and thereby support the rehabilitation process.

Mobile technology has successfully been applied to fulfill various functions related to rehabilitation among people with dementia or other neurological diagnoses, such as providing memory support following traumatic brain injury [11]; monitoring activity, mobility [12,13], and goal setting [14]; and for rehabilitation after stroke [14]. We build upon this work by extending and combining support and monitoring capabilities of personal devices and by implementing this among the target population of people with dementia.

### Aim and Objectives

The primary aim of this work is to evaluate the feasibility of using mobile and wearable technology to support rehabilitation for dementia. We have implemented a technological setup combining both personalized support and behavioral monitoring among people with dementia in a real-life context in a series of 6 in-depth case studies to address three main objectives:

1. Examine the technical feasibility of sensor-based behavioral measurement using smartphones and smartwatches among people with dementia.
2. Compare participants’ subjective perceptions of their behavior with objective, sensor-derived measures.
3. Evaluate user acceptance focusing on usefulness and health efficacy.

Through fulfilling these objectives, this work will contribute new evidence to the potential for smartphones and wearables to benefit rehabilitation in practice, identifying areas for further research on a larger scale. Addressing new opportunities presented by mobile devices and the data these generate makes an important step toward the wider vision of data-driven health care interventions that enable predictive, preventative, personalized, and participatory (P4) health care [15,16].

## Methods

### Study Design

**Figure 1:**
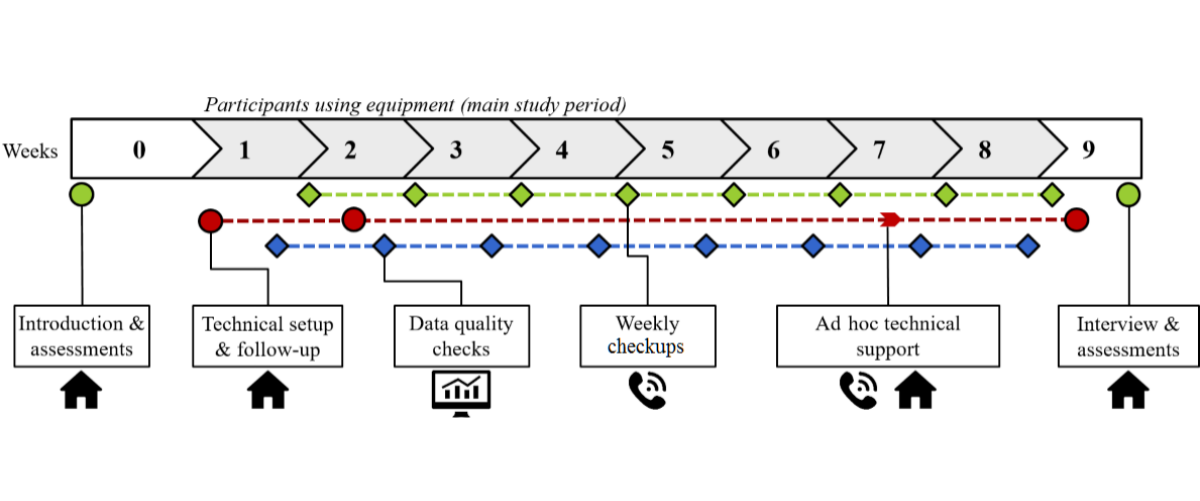
Procedure for each case study showing interaction points and data collection.

A longitudinal study design comprising 6 case studies was employed. Each case included both a main participant (ie, person with dementia) and their caregiver over a period of at least 8 weeks. During the study, participants used smart devices for support in everyday life, answered daily reports on their mobility and activity levels, and followed an individual goal. Behavioral data was recorded from device sensors throughout participation. The study procedure is outlined in Figure 1, which shows the series of participant interactions through which both quantitative and qualitative data were collected throughout the process.

### Recruitment and Participants

Recruitment was carried out through the dementia and memory clinic at a Danish hospital. The population of interest is community-dwelling adults in the early stages of dementia, which includes people with mild-to-moderate cognitive impairment. Participants were required to live with their primary caregiver, due to the supportive role the caregiver plays in the study (eg, providing information). Participants were not required to have any prior experience using smart technology. Exclusion criteria included any disability that would either affect the participant’s ability to use smart technology (eg, severe vision or hearing loss and apraxia) or cause extremely limited mobility and activity levels (eg, home- or bed-bound participants).

A total of 6 participants—2 female (33%) and 4 male (67%)—between 65 and 78 years of age completed the study. A further 3 participants who enrolled dropped out due to illness or feeling daunted at the prospect of using the devices. A summary of the participants’ demographic and clinical backgrounds is provided in Table 1. Cognitive assessment scores were collected from the clinic through which participants were recruited. While certain scores were within the normal range for cognitive function, behavioral and executive symptoms in the early stages of dementia were not always captured by the Mini-Mental State Examination (MMSE) and Addenbrooke's Cognitive Examination (ACE). All participants were diagnosed with mild-to-moderate cognitive impairment based on specialized evaluation at the clinic. A brief introduction to the participants in each case study is provided in the Results section.

**Table 1:** Summary of participant demographics and assessment scores.

Participant information, measure			Participant					
			1	2	3	4	5	6
Age in years			78	70	65	68	70	67
Years retired			16	2	3	2	10	2
Gender			Male	Male	Female	Male	Female	Male
**Cognitive impairment**								
	MMSE^a^		27	27	24	26	27	23
	ACE^b^		75	75	71	77	88	81
**Quality of life**								
	**QoL-AD^c^**							
		1^d^	33	44	41	51	45	50
		2^e^	38	42	46	49	49	48
**Functional performance**								
	**FAQ-IADL^f^**							
		1	4	5	10	0	5	10
		2	6	9	5	0	5	12
**Caregiver burden**								
	**ZBI^g^**							
		1	11	26	0	N/A^h^	N/A	20
		2	1	28	0	18	3	13
**Mobility**								
	**LSA^i^**							
		1	76	84	50	66	100	66
		2	74	84	84	90	84	74
**Activity**								
	**GPAQ^j^ (hours/week)**							
		1	27.50	10.00	20.00	39.50	70.25	7.00
		2	37.50	14.00	34.75	32.75	37.00	14.50

^a^MMSE: Mini-Mental State Examination.

^b^ACE: Addenbrooke's Cognitive Examination.

^c^QoL-AD: Quality of Life in Alzheimer's Disease scale.

^d^Prestudy measurements.

^e^Poststudy measurements.

^f^FAQ-IADL: Functional Assessment Questionnaire-Instrumental Activities of Daily Living.

^g^ZBI: Zarit Burden Interview.

^h^N/A: not applicable.

^i^LSA: Life-Space Assessment. The LSA, adapted, was used: the score was calculated assuming independent travel.

^j^GPAQ: Global Physical Activity Questionnaire. Results show total active time for work, including household, leisure, and transport (ie, walking or cycling).

### Technical Setup for Support and Monitoring

**Figure 2:**
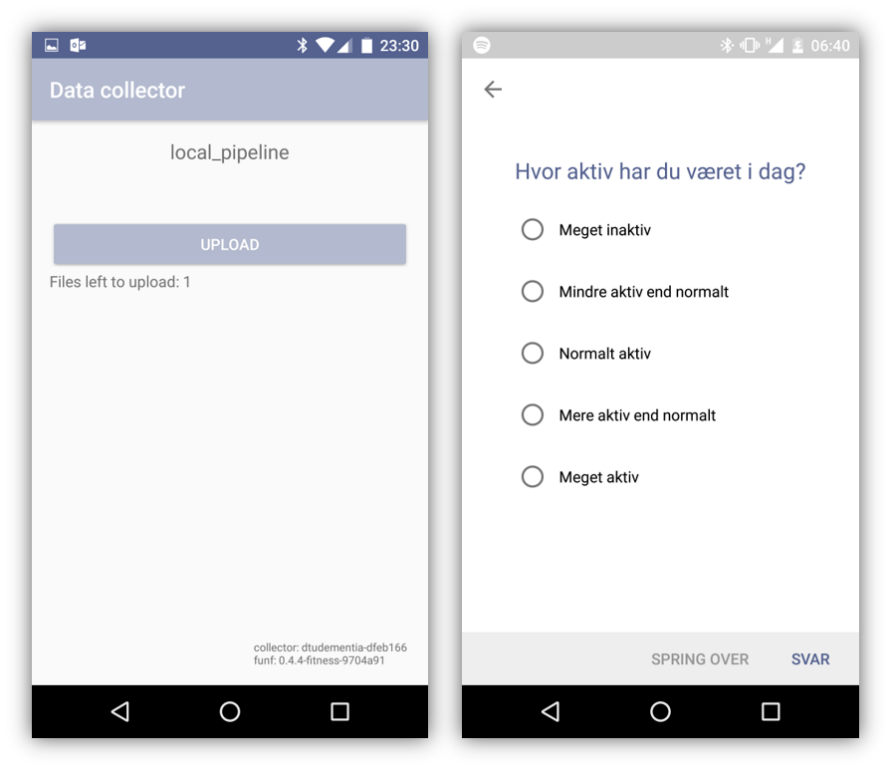
The data collector custom app (left) runs in the background collecting device sensor and log data. The mobile self-reporting app (right) prompts users to evaluate their daily activity and mobility levels.

Mobile devices were employed to support participants in their everyday lives and to collect behavioral data. The technological setup comprised a smartphone (ie, Nexus 5 running Android OS v6.0.1.) paired with a smartwatch (ie, Sony SmartWatch 3 running Android Wear), a mobile self-reporting module, and an app for secure collection of sensor data and logs from the devices (see Figure 2). A detailed description of the system is available in Thorpe et al [17].

A technical orientation meeting was held at the start of each case study to introduce the devices and to personalize device setup in collaboration with the participant and their caregiver. As a standard setup, the smartphone home screen displayed the time; upcoming appointments; and, based on interest from all participants, a daily step count. All participants were provided with Google Calendar and shown how this could be used to remember tasks or appointments, since this was shown to be useful and not too challenging for participants in an earlier study [18]. This standard setup was then further adapted by adjusting settings or adding apps to fit each participant according to individual needs, including what they already used. A follow-up visit 1 week after orientation was carried out to resolve early issues and repeat instructions. Participants were also provided with an illustrated manual and offered technical support throughout the study over the phone and during in-home visits, as required.

For collection of behavioral data, two custom apps were deployed: (1) a data collector app that runs in the background recording activity and location data from device sensors and (2) a self-reporting app that prompts input from users regarding their perceived mobility and activity according to a predefined schedule.

### Goal Setting and Following

Participants set individual goals together with a member of the research team trained in psychology, which they followed throughout participation. Goal setting is used extensively in rehabilitation and was, therefore, emulated in the case studies specifically within the themes of mobility and activity and according to participants’ own views of what was important to their lifestyle.

Goals included getting out of the house each day (participants 3 and 6), walking longer daily (participant 2), driving less and riding a bicycle more (participant 4), and maintaining an existing activity schedule (participants 1 and 5). Cognitive impairment can affect a person’s ability to function in everyday life and remain socially engaged, leading to depressive symptoms and reclusiveness that can further aggravate cognitive decline. Such mobility- and activity-related goals are, therefore, intended to encourage participants to remain active and engaged, eliciting support as required to achieve their goals. Participants were asked to evaluate their own goal attainment in weekly phone calls, which provided an opportunity for them to further qualify their answers.

### Data Collection

Quantitative and qualitative data were collected throughout the studies, as outlined in Table 2 and in Figure 1 showing the protocol steps.

#### Background and Assessments

Background information and most recent cognitive impairment test results were collected at the start of the study from the clinic. A set of questionnaire-based assessments was performed at the start and end of participation. These were collected as a reference for participants’ profiles and in the case of marked change that may influence the participants’ experiences; they were not used for pre-post analyses. Questionnaires included assessments for quality of life, function in daily living activities, caregiver burden, and mobility and activity (see Table 2). Mobility and activity questionnaires were adapted to fit the study purposes; independence level was excluded from the Life-Space Assessment (LSA) used to measure mobility [19], and activity intensity grading was excluded from the sections selected from the Global Physical Activity Questionnaire (GPAQ) [20].

**Table 2:** Summary of data collected from the case studies.

Category of data, frequency of data collection		Description
**Background information**		
	At study start	Demographic: age, gender, education, and occupation Cognitive impairment severity: MMSE^a^ and ACE^b^
**Questionnaires**		
	At study start and end	Quality of life: QoL-AD^c^ Functional performance: FAQ-IADL^d^ Caregiver burden: ZBI^e^, short form Mobility: LSA^f^, adapted Activity: GPAQ^g^, adapted
**Device data**		
	Continuous	Location Activity Step count Battery status Screen on and off
**Mobile self-reports**		
	Daily (evening)	Perceived mobility (daily) Perceived activity level (daily)
**Meetings and interviews**		
	Weekly	Phone calls: perceived goal attainment score and supplementary notes
	At study end	Semistructured interview on experiences and outcomes
	Ad hoc	Support interactions

^a^MMSE: Mini-Mental State Examination.

^b^ACE: Addenbrooke's Cognitive Examination.

^c^QoL-AD: Quality of Life in Alzheimer's Disease scale.

^d^FAQ-IADL: Functional Assessment Questionnaire-Instrumental Activities of Daily Living.

^e^ZBI: Zarit Burden Interview.

^f^LSA: Life-Space Assessment.

^g^GPAQ: Global Physical Activity Questionnaire.

#### Sensor Data From Mobile Devices

Data from device sensors and logs were collected throughout study participation. Location and activity data, including steps and recognized activities, were recorded and used to calculate a set of mobility and activity metrics. The set of algorithms used to measure mobility and activity behavior was described in detail in an earlier study by Thorpe et al [17]; this set combined a range of metrics used for similar monitoring purposes in related literature [21-24].

Location data merged from the phone and watch was first used to extract a series of stays and moves throughout each day. These, together with the raw global positioning system (GPS) coordinates, were then used to calculate a set of spatial, temporal, and frequency-based mobility measures, including the following:

1. Minimum convex polygon (MCP) or mobility envelope (ie, area of the polygon constructed around location data).
2. Action range or home range (ie, furthest straight-line distance travelled).
3. Total distance covered, out of home only.
4. Time spent out of home.
5. Time spent moving between locations.
6. Number of places visited.
7. Number of trips.

Recognized activities from the phone only, which were accessed using Google’s activity recognition application programming interface, were used to extract bouts of activity within the categories: still, on foot, bicycle, and vehicle. Total daily steps were recorded independently from the phone and watch. This data were used to calculate the following activity measures:

1. Active time: sum of durations of all activity bouts on foot and bicycle.
2. Active bouts: total count of all activity bouts on foot and bicycle.
3. Still time: sum of duration of still bouts.
4. Total steps.

#### Self-Reported Activity, Mobility, and Goal Attainment

Mobile self-reports were issued daily asking participants about their perceived activity and mobility that day relative to their normal daily level on a 5-point scale. The five responses were as follows: *much less than normal*, *less than normal*, *normal*, *more than normal*, or *much more than normal*. These were recorded as values within the set {0, 0.25, 0.5, 0.75, 1}, where 0.5 equates to *normal* for interpretation as a median.

Participants evaluated their own goal attainment weekly in a regular phone call. Goal attainment was scored along a range from -2 to 2, where a score of 0 was assigned if the goal is met, with 2 levels in either direction for under- or overachievement, as has been described by Turner-Stokes [25].

#### Qualitative Data From Participant Interactions and Semistructured Interviews

A semistructured interview with each participant at the end of the study was used to gather qualitative data on their experiences, particularly in relation to technology acceptance factors. Interviews included questions on the following:

1. Pre-existing tools and coping strategies.
2. Experiences using the technology, including how it was used and for which purposes as well as the difficulties and benefits of its use.
3. Adequacy of the technical support and participants’ own knowledge and skills for operating the devices.
4. Expectations and whether these were met, which needs were not met, and any desired but absent functionality.
5. Impact on participants’ everyday lives and on their health.

Notes from the weekly phone calls as well as technical support logs were also collected.

### Data Analysis

#### Overview

Three main analyses were performed to fulfill the study objectives. The first examines the availability and utility of the data generated by the smart devices; the second examines agreement between participants’ subjective reports of their behaviors and objectives with sensor-based measures; and the third examines user acceptance factors.

#### Analysis 1: Data Availability and Utility

The aim of this analysis was to determine whether the smart devices, as used in a real-life context, generate adequate data for the intended purpose of monitoring behavior. This depends both on the technology functioning correctly and on the participants using them sufficiently (ie, keeping the devices charged and connected, carrying them around with them, and answering mobile self-reports). We examined the following:

1. Data availability: the proportion of the study period for which data were available from each of the devices and from the mobile self-reports.
2. Data utility: visual inspection of the device interaction and behavioral data to evaluate whether data quality and quantity were sufficient for behavioral patterns to be evident.

#### Analysis 2: Self-Reported Versus Sensor-Based Measurement of Behavior

#### Overview

The aim of this analysis was to determine whether the behavioral insights gained from sensor data agree with the participants’ own perceptions of their behavior. Since current rehabilitation approaches rely heavily on input from participants and caregivers to assess progress and outcomes, it is also relevant to investigate how such subjective reports differ from the proposed sensor-based approach. Identifying where these differ can guide further investigation into whether this is due to inaccurate perceptions or technical failures. Sensor-derived measures were compared with participants’ mobile self-reports of activity and mobility levels and their weekly goal attainment ratings.

#### Daily Activity and Mobility

Sensor-based measures of activity and mobility vary in units of measurement and were, therefore, ranked as percentiles using the empirical cumulative distribution function for comparison with self-reports (5-point scale). The self-reported and sensor-based measures were then plotted together for comparison within each case study.

#### Weekly Goal Attainment

The comparison between perceived goal attainment and sensor-based measures required individual analysis according to each participant’s defined goals. In most cases, this involved selecting relevant activity and/or mobility measures and aggregating these by week. Other methods included detecting visits to a specified location (eg, a training center), which required a priori knowledge of the location’s GPS coordinates. Goal attainment ratings ranged from -2 to 2, where 0 corresponded to having met the goal; there were 2 levels for under- or overachievement in either direction. Following the ranking of sensor-based measures as percentiles, as in the comparison with mobile self-reports, these were shifted by 0.5 such that the median lay on the zero line for comparison with goal attainment ratings.

#### Analysis 3: Qualitative Analysis of User Acceptance—Usefulness and Health Efficacy

The aim of the third analysis was to evaluate potential acceptance of the devices for support. Usability and usefulness are two important factors influencing technology acceptance, as proposed in the technology adoption model and numerous adaptations thereof [26]. We were interested in whether participants benefited from support selected from a broad range of available tools; therefore, usefulness was more appropriate than usability, which was evaluated for a similar smartphone and smartwatch setup among the population of interest in Thorpe et al [18]. Within health care technology, health efficacy is a further important consideration [27]. This analysis, therefore, focused on usefulness and health efficacy in terms of impact on aspects of quality of life, such as function in everyday life and social engagement. Interview recordings were transcribed and analyzed to identify the following:

1. Support offered to the participants by the mobile devices, the extent to which this was beneficial, and which support needs were met.
2. Perceived impact of using the technology on aspects of quality of life, such as function, independence, behavior, mood, or social engagement.

## Results

### Overview

Results are presented for three analyses on the following: data availability and utility, comparison between sensor-based and self-reported behavioral measurement, and user acceptance. As further background, a brief introduction to the 6 participants in the case studies is provided in Textbox 1.

**Brief introduction to the 6 case study participants.**

*Participant 1* (male, 78 years) lives with his wife and son. He has been retired for 16 years at the time of participation but follows an active weekly schedule involving diverse sports, hobbies, and interests that he often travels to by bicycle. This is the only participant who used his own smartphone for the study; other participants already using smartphones owned iPhones, which were incompatible with the custom apps for data collection.

*Participant 2* (male, 70 years) lives with his wife, whom he depends on heavily for support in everyday life. She manages his schedule and they agree that she is “his most important aid.” He struggles with fatigue and low energy, some days sleeping most of the day, but enjoys going out for walks when able. He owns an iPhone and, though he could not get used to the study phone, was able to operate the devices adequately for completing the study.

*Participant 3* (65 years, female) lives with her husband. She has battled with psychosocial consequences of symptoms, such as anxiety and feeling unsafe leaving the house due to fear of not finding her way home. She keeps physically active doing housework, going out for walks, and doing physical training at home. She has used an iPhone but successfully learned to use the study phone to the same extent.

*Participant 4* (male, 68 years) lives with his cohabiting partner. He describes the greatest impact his diagnosis has had as being the loss of his driver’s license, which he had regained for 2 years just prior to participation. He keeps active by taking his dogs out, visiting a local center aimed at retired members of the community, and riding his bicycle. He also enjoys hobbies such as gardening and playing music. While he owns an iPhone that he uses extensively, he describes a disinterest in technology generally. He did not use the study phone for anything further than pairing it to the watch and answering mobile self-reports, as he did not feel comfortable learning to use a new device.

*Participant 5* (female, 70 years) lives with her husband and has been retired for 10 years. She is highly active, with a physical training schedule including sessions 5 days per week. She is not experienced with smart technology and, though they own a household iPhone, she prefers to use a basic Nokia. She, therefore, did not use the study phone for other functions besides answering the mobile self-reports. She found the watch uncomfortable and did not use it for the study. A decrease in motivation during the study was reported and attributed to psychosomatic symptoms (ie, pain and fatigue).

*Participant 6* (male, 67 years) lives with his wife. He retired earlier than planned due to health complications and tries to keep active and engaged by doing light housework, shopping, or going for walks. He has no prior experience with a smartphone. He has a basic Nokia that he can use for calls and for reading text messages but not for writing them. Despite his limited experience, he did not feel it was too difficult to use the devices for the basic purposes required (eg, turning them on and off and reading his step count) but needed help from his wife answering mobile self-reports.

### Analysis 1: Data Availability and Utility

Here we examine whether adequate data were generated for the intended purpose of monitoring behavior among the population of interest. Table 3 shows the duration in days for participants in each case study along with how many days data were recorded at all, from the smartwatch, and from mobile self-reports. For mobile self-reports, a value of 0.5 was assigned for each of the two questions per day.

While data were recorded on nearly all days, only a small proportion was generated by the watch. Participant 5 did not wear the watch. Several participants had difficulty maintaining the connection between the watch and phone, including noticing when they were disconnected. A further known cause was a technical fault, whereby watch data were not transferred to the phone. This necessitated a manual upload and reset of both devices, which took days or weeks to resolve depending on the participants’ availability. Missing self-reports were mostly attributed to technical failures and usability issues rather than adherence. Participant 1 only started receiving the questions 7 weeks into the study and, consequently, agreed to extend his participation. Participant 6 could not answer self-reports without assistance from his caregiver. Limited availability of watch data and self-reports did not necessarily inhibit the potential for behavioral monitoring in practice; all data types were recorded from the phone sensors, from which data were available throughout the study, and self-reports were for research purposes only. One noteworthy limitation was regarding step count, which was expected to be more reliable from the worn watch than from a phone that needed to be carried.

**Figure 3:**
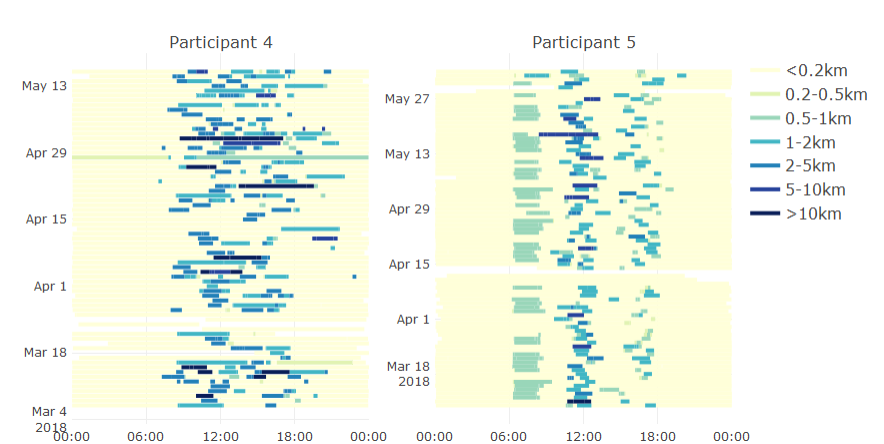
Location data for 2 participants throughout the study period. The time of day is shown along the horizontal axis and increasing date is shown along the vertical axis.

**Figure 4:**
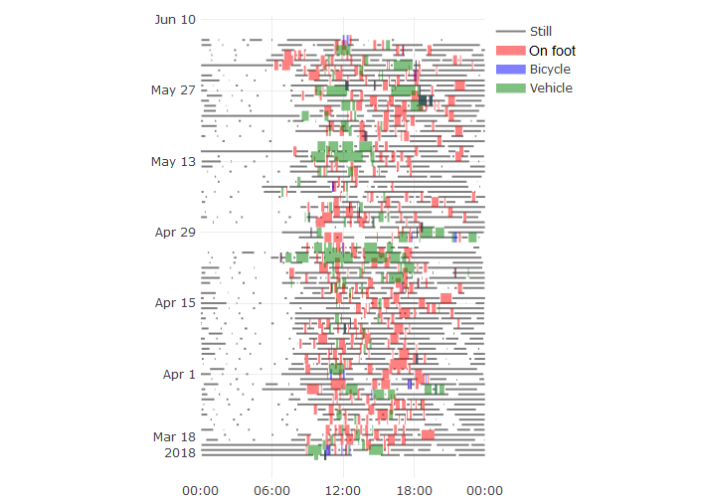
Activity data for participant 2 throughout the study period. The time of day is shown along the horizontal axis and increasing date is shown along the vertical axis.

Visual inspection of the generated data indicates that this could indeed be adequate for behavioral monitoring. Figure 3 shows location data generated throughout the study for participants 4 and 5, indicating distance from home as a color scale. These demonstrate that the devices were carried with them and show the potential for resulting data to reveal patterns in behavior. For example, a daily rhythm is evident for participant 5 (ie, movement from 6 am to 8 am near home, further movement around noon, and less-consistent activity in the late afternoon), which contrasts with the less-structured movement for participant 4. Figure 4 shows activity data for participant 2, again showing that the phone was used enough to generate activity bouts throughout the study. This figure reveals the habit of walking during the evenings that the participant reported having started during the study out of motivation to increase his daily step count.

**Figure 5:**
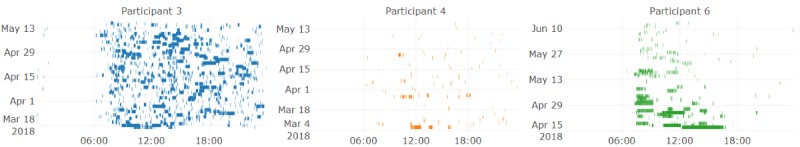
Screen-on time for 3 participants over their entire participation period. The time of day is shown along the horizontal axis and increasing date is shown along the vertical axis.

The three examples above are representative of all cases in terms of availability of data. This was sufficient despite variation in levels of interaction with the devices. Figure 5 shows screen-on time as an indication of device interaction for participant 3 who used the phone, participant 4 who did not use the phone, and participant 6 who appears to have attempted to use the phone at the start, with interaction diminishing over time.

**Table 3:** Data availability.

Participant	Participant duration (days), N	Data recorded at all (days), n (%)	Data recorded by smartwatch (days), n (%)	Data recorded by self-reports (days), n (%)
1	98	98 (100)	57 (58)	39.0 (40)
2	82	81 (99)	4 (5)	59.5 (73)
3	60	60 (100)	10 (17)	53.5 (89)
4	72	72 (100)	8 (11)	39.0 (54)
5	87	86 (99)	0 (0)	49.5 (57)
6	57	54 (95)	2 (4)	46.0 (81)

### Analysis 2: Self-Reported Versus Sensor-Based Measurement of Behavior

#### Daily Activity and Mobility

Self-reported daily levels of mobility and activity were compared with sensor-derived measures. Measures were selected for analysis from the complete set based on several factors. A total of 4 participants out of 6 (67%) traveled out of town during participation, which affected measures such as *action range* or *time out of home* for all days away. For mobility, the measures *MCP*, *time spent moving*, and *number of places visited* were therefore included, which were only elevated on the actual days of travel. For activity, the measures *active time*, *active bouts*, and *steps* were included. Steps were taken only from the phone due to the limited watch data and potential for large differences in the count from different sources that could skew results if combined. A combined measure for mobility and activity was calculated as the average of the three measures for each.

**Figure 6:**
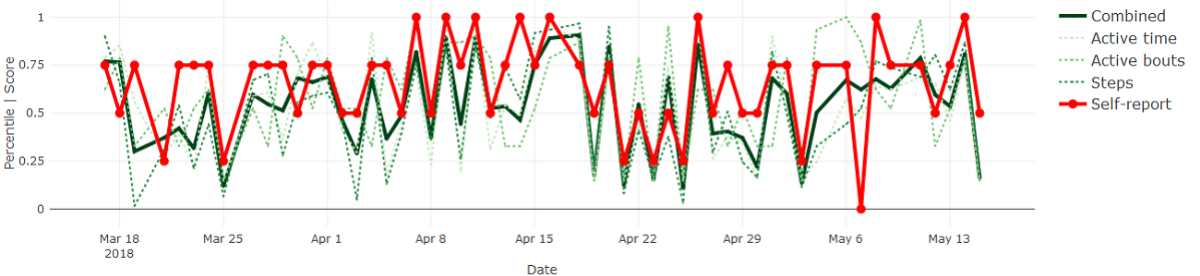
Activity measures and self-reports for participant 3.

**Figure 7:**
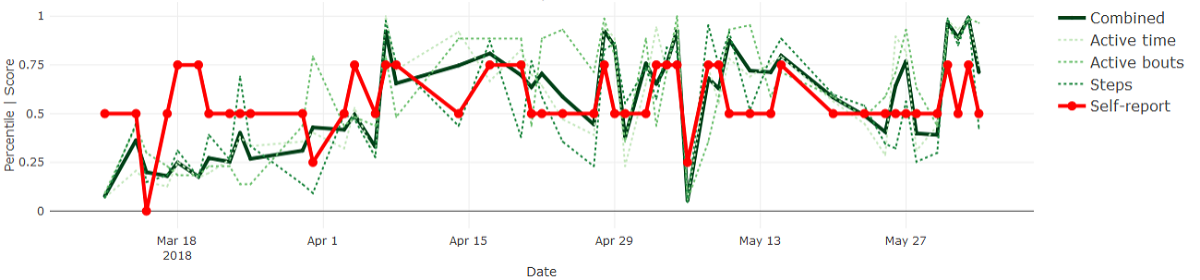
Activity measures and self-reports for participant 5.

**Figure 8:**
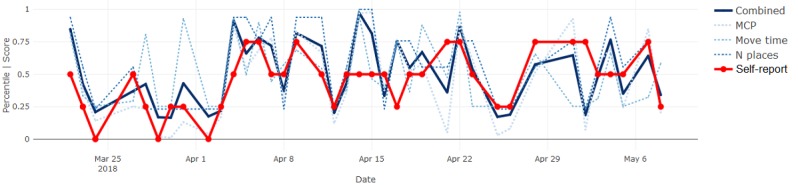
Mobility measures and self-reports for participant 1. MCP: minimum convex polygon.

**Figure 9:**
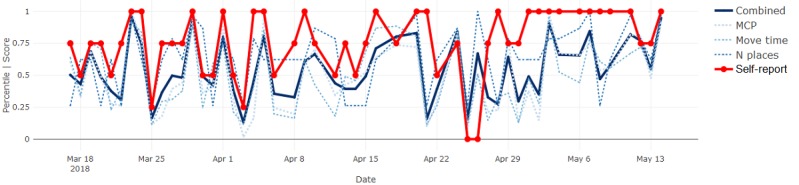
Mobility measures and self-reports for participant 3. MCP: minimum convex polygon.

By definition, most days were expected to be reported as *normal* and show sensor measurements near the median, inevitably resulting in agreement between the signals. Therefore, we focused on deviations from normal to determine whether the sensor-based measures followed subjectively reported fluctuations in behavior. Where they did not, we proposed sources of such disagreement and inferred potential advantages or disadvantages of each modality. Four illustrative results are included in Figures 6-9.

Figure 6 shows that the sensor-based measures closely aligned with frequent (ie, day-to-day) fluctuations in perceived activity levels for participant 3, whereas a slower trend (ie, increase over the first month) in the sensor-based measures for participant 5 was not reflected in her self-reported measures (see Figure 7). This could indicate that changes that were more gradual were not as easily perceived by the individual.

The mobility measures for participant 1 (see Figure 8) again show alignment in the direction of fluctuations, only this time with some distinct deviations, most notably in mid-April. This participant felt unsure about how to describe his mobility in relation to his schedule, noting that “normal for a Tuesday is different from normal for a Friday.” This could explain why higher mobility might have been reported as normal, requiring careful consideration of seasonality for detecting deviations in behavioral signals.

In the mobility comparison for participant 3 (see Figure 9), subjective reports lay predominantly in the upper range (ie, above normal) and were, thus, consistently higher than the sensor-based data. This participant described the substantial impact that the smartphone-based support had on her mobility and showed an increase in both mobility and activity assessment results from pre- to poststudy. Therefore, it is possible that these would be reasonable perceptions given a longer period. Alternatively, the positive impact on her mood and confidence could have exaggerated her perception of her own mobility. Other possible sources of disagreement, particularly for isolated deviations, were mistakes in using the mobile self-reports and forgetting to carry the phone.

A final observation from the results is that the combined measures appeared to agree better with participants’ perceptions than each on their own. These were also potentially more robust against algorithm errors (eg, misclassification of activities or travel trajectories).

#### Weekly Goal Attainment

#### Overview

Participants’ goals can be grouped into three categories:

1. Target frequency of getting out of the home.
2. Increase or decrease in an activity type.
3. Adherence to an activity schedule.

Here we use examples from each category to examine whether sensor-based measures could be used to evaluate goal attainment, comparing these with participants’ perceptions.

#### Target Frequency of Getting Out of the Home

**Figure 10:**
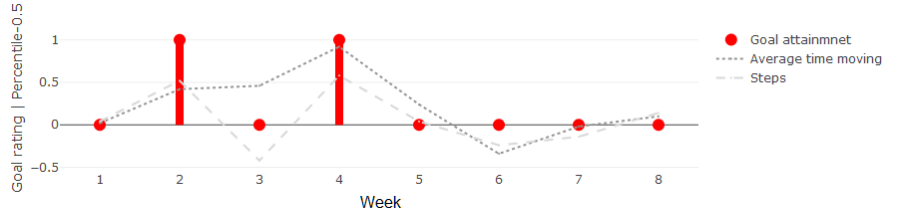
Weekly goal attainment (red) and relevant average weekly measures (ie, shifted cumulative distribution function probabilities) for participant 6.

Participants 3 and 6 followed the goal of getting out of their homes every day. This can be measured directly from the location data as the number of days per week that the participant went out at least once. Both participants went out 6-7 days per week during most of the study and each rated their goal as achieved or overachieved (score of 0 or 1) on those weeks. Participant 3 reported 1 week of underachieving the goal (score of -1), which was also the only week she went out for only 5 days, the minimum for her participation period. Participant 6 reported overachievement twice. While these weeks did not show any noticeable difference in number of days per week that he got out, they did show higher-than-usual step counts and time spent out moving between stays. This is demonstrated in Figure 10, which shows the average for these behavioral measures over each week along with goal attainment ratings.

#### Increase and Decrease in an Activity Type

**Figure 11:**
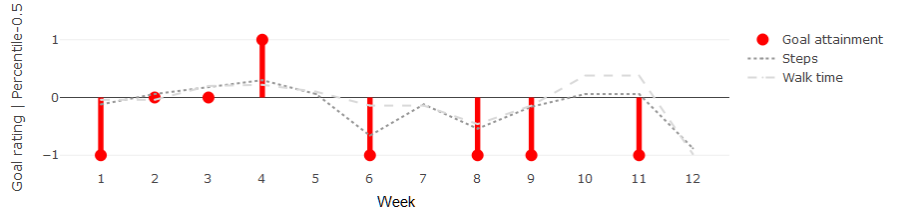
Weekly goal attainment (red) and relevant average weekly measures (ie, shifted cumulative distribution function probabilities) for participant 2.

A total of 2 participants out of 6 (33%) defined goals of increasing an activity type: participant 2 aimed to walk more and participant 4 aimed to cycle more and drive less. This can be calculated from the extracted activity bouts as the amount of time spent in the relevant activity. For walking, step count was also included. Figure 11 shows the weekly goal attainment scores for participant 2 along with weekly averages for total daily steps and time spent on foot. The figure shows that the sensor-based measures fell under the median on those weeks where the participant perceived underachievement, over the median for overachievement, and close to the median for achievement. This indicates that there was agreement between the sensor-based measures and the participant’s perceptions, should goal achievement correspond to normal behavior.

A similar approach was used to measure bicycle and vehicle activity for participant 4. However, when rating his goal attainment, he usually explained that he had not been riding his bike and discussed other activities instead. Around halfway through his participation, he first reported having started to ride his bicycle. This transition is evident in Table 4, which shows a marked increase in the total time spent cycling per week from the point at which he first reported cycling (week 5). The cycling time prior to week 5 was quite possibly the result of misclassifications in the activity recognition from Google, since confusion between vehicle and bicycle activities is a known issue [17].

**Table 4:** Weekly goal attainment for participant 4.

Participant	Participant duration (days), N	Data recorded at all (days), n (%)	Data recorded by smartwatch (days), n (%)	Data recorded by self-reports (days), n (%)
1	98	98 (100)	57 (58)	39.0 (40)
2	82	81 (99)	4 (5)	59.5 (73)
3	60	60 (100)	10 (17)	53.5 (89)
4	72	72 (100)	8 (11)	39.0 (54)
5	87	86 (99)	0 (0)	49.5 (57)
6	57	54 (95)	2 (4)	46.0 (81)

^a^Missing information.

#### Adherence to an Activity Schedule

Activity schedule goals were detected using information provided by the relevant participants about their schedules and locations. Participant 5 trained 5 days per week at a training center, for which the location was provided. Visits to the center were detected based on distances between its location and the centroids of all stay events in the dataset. A summary of the number of days per week that she visited the training center and her reported goal attainment scores is provided in Table 5, which shows little agreement between her perceptions and sensor-based measures. The notes from weekly calls offer some explanation for the disconnect: her answers tended to be substantiated by how active she was, generally, rather than her training schedule, with reference to long walks or step counts. Furthermore, she twice rated her goal as achieved with an explanation that the training center was closed due to holidays, suggesting that since this was beyond her control and she remained active nevertheless, it was not perceived as underachievement on her part.

Generally, while in most cases a single sensor-based measurement of the goal did not correspond to participants’ ratings, several related measures could sufficiently describe the behavior motivating these. This encourages the use of a multimodal approach to sensor-based monitoring. In the next analysis, we shift focus from monitoring to support from the devices for people with dementia.

**Table 5:** Weekly goal attainment for participant 5.

Goal information	Week							
	1	2	3	4	5	6	7	8
Reported goal attainment, score	-1	-1	0	X^a^	-1	0	0	0
Total time cycling, minutes	23	0	26	12	78	65	50	40

^a^Goal rating of 0 is *achieved*, -1 is *underachieved*, and 1 is *overachieved*.

### Analysis 3: Qualitative Analysis of User Acceptance—Usefulness and Health Efficacy

#### Overview

Here we present results from the qualitative analysis of interview data to evaluate how useful support from the devices was and the impact this support had on quality of life.

Pre-existing support from smartphones was taken into consideration and mostly continued alongside the personalized support offered in the studies. Participants 1, 2, 3, and 4 used smartphones prior to the study and described using their smartphone daily or carrying it on them. Caregivers of participants 1 and 4 remarked on the dependence their spouse had on their phone, explaining that it is their “lifeblood” (participant 4) and how they might interrupt a meal to capture information on their phone before forgetting it (participant 1). Examples of purposes given for which the phones were used included calendar and reminders, taking and reviewing pictures, social, leisure, news and weather information apps, and to make payments.

#### Usefulness

Results from the analysis of poststudy interviews regarding usefulness of the personalized support offered to participants are summarized in Table 6. Interview recordings were analyzed to extract information about how the participants benefited from the support and to what extent. The same app might fulfill different purposes for different participants. For example, a location tracker was used by participant 5 to review her routes after a journey for fun (ie, leisure) and to remember where she has been (ie, memory); the same tracker was used by participant 3’s caregiver to locate her (ie, safety). The picture-dialing feature, whereby participants added pictures to contact details in their phonebooks, offered memory support to participant 1, whereas participant 2 reported feeling safer when the phone rang if a picture was shown.

**Table 6:** Personalized support offered to participants based on individual needs.

Goal information	Week							
	1	2	6	7	8	9	10	11
Goal rating^a^, score	0	1	0	0	-1	1	0	0
Training days	5	5	4	5	4	4	5	2

^a^Responses are shown with + or - to indicate positive or negative perceptions of benefit from the support, respectively; stronger positive or negative responses are indicated with ++ or --, respectively.

Responses to the smartwatch varied substantially. A total of 2 out of 6 participants (33%) did not like it at all; participant 5 explained that “it's big and it's heavy, and I must take care of it and have to take it everywhere with me,” and participant 6 found the rubber strap to be uncomfortable, especially in warm weather. Other participants found the smartwatch enjoyable or convenient; participant 4 added that he did not notice it at all and preferred it to checking the time on his phone.

Overall, the support was perceived as beneficial. Two cases stand out as being particularly negative or positive:

1. Participant 1 was provided with Google Fit based on an interest in information about his activity; however, he misunderstood that he should manually enter all of his activities into Google Fit as a means of data collection in the study. This caused considerable effort on his part, led to anxiety over mistakes in recording the activities, and was challenging to clarify and resolve.
2. Participant 3’s experience was overwhelmingly positive. She described her participation in the study as having “given her a new life,” primarily attributed to personalized support selected to help her navigate home and for her caregiver (ie, husband) to track her location. She explains that “I have a new life... my husband is completely calm... Can you imagine, I can go anywhere! ... [with the find home feature] I have peace of mind and inner calm.” Her caregiver describes no longer being scared of her getting lost and notes the impact on her: “We [family members] can indeed notice a huge difference, really. She is completely changed. She has become super positive, and has her good humor back.”

#### Health Efficacy

Participants had varying views on the impact that the technology and broader intervention had on their everyday functioning, health, and well-being. Participants 1 and 5 perceived no benefits, both stating that they were active to begin with and were satisfied with their existing coping strategies. Participants 2, 4, and 6 all reported that they found tracking their step count to be motivating; participants 2 and 6 believed they had increased their activity as a result; for both, their pre- and postactivity assessments also showed an increase. Participant 3 perceived considerable impact on both functioning in everyday life and health status, reporting reduced anxiety, improved mood, increased activity levels, and having implemented more effective schedule management using the study devices.

To assess overall acceptance, participants were asked whether they would be interested in using the support beyond the study. Only participant 5, who had no previous experience with smartphones, expressed no interest at all; she explained that the benefits were not worth the effort and she felt satisfied with her pre-existing coping strategies. For participants who used smartphones prior to the study, interest was mostly dependent on being able to implement the setup with their own devices, with the exception of participant 3 who wished to continue with the study setup. All participants, including those without a personal interest in using the technology, felt that it could benefit other people with dementia.

## Discussion

### Principal Findings

This work describes 6 in-depth case studies to evaluate the feasibility of using mobile and wearable technology (ie, smartphones and smartwatches) to support rehabilitation among people with dementia. Results demonstrated the potential for data from personal devices used in a real-life context among the target population to reveal behavioral patterns, even with limited device interaction. Comparisons between objective, sensor-based measurement and participants’ self-reports showed that sensor-based measures of mobility and activity reflected participants’ perceived fluctuations in behavior, may reveal gradual trends not detected by the participants themselves, and provided insight into goal attainment for a range of related goals. Qualitative analysis of user acceptance indicated that personalized support offered by smart technology addressed functional, memory, safety, leisure, and psychosocial needs, where many of these depend on familiarity of the device and platform for user acceptance. Of 6 participants, 4 (67%) perceived this support to positively impact their health, mostly regarding motivation to be active, with 1 participant further describing considerable positive impact on perceived anxiety, independence, activity, and caregiver burden.

These findings demonstrate potential for smartphones and wearables to offer pervasive support that could benefit people with dementia, while also generating rich data to monitor behavior for evaluating function and engagement.

### Implications for Clinical Practice

Here we discuss several implications of the findings of this research, particularly for implementation in practice.

#### Remote Behavioral Monitoring

This study shows high data availability from smartphones used in a real-world setting by the target population, including for low device interaction. This indicates that a sensor-based behavioral monitoring approach is not restricted to users inclined toward higher smartphone use, thus broadening the potential target group.

The data were used to derive a set of mobility and activity measures comparable to participants’ own perceptions of their behavior. This result implies that the monitoring approach could replace traditional methods for gathering information about behavior (eg, questionnaires or interviews) with several advantages:

1. Scaled-up data output provides higher-resolution behavioral patterns and insight into day-to-day changes in behavior to enable more predictive and preventative care strategies.
2. Remote, passive measurement reduces burden on both the health care professional and care recipient and is, therefore, less resource-heavy than traditional approaches.
3. Data quality is not dependent on the user’s ability to recall information.
4. Availability of objective information that can be visualized and shared between the care recipient and provider can facilitate collaborative care.

#### Goal Setting and Following

The use of goals in rehabilitation is well established [4,28]; however, this raised several challenges in this study. A total of 2 participants who were satisfied with their current lifestyle followed goals to maintain their schedule. While such a goal is relevant for people with dementia due a risk of decline in functional capacity, it was sometimes difficult for these participants to conceptualize or feel motivated by a goal to maintain rather than improve upon their status quo. Participants also generally found it difficult to recall their goal over the duration of the study, in some cases providing irrelevant information when asked about goal attainment.

This study indicates that sensor-based behavioral measures could aid in the following of goals by generating rich insights into behavior related to individual goals. This information could provide feedback regarding goal attainment to make it easier for care recipients to recall and follow goals and to motivate goal attainment.

#### Support in Everyday Life

Smartphones were found to be a feasible platform for offering support in everyday life. Indeed, most participants already used their own smartphones extensively as memory support, among other purposes, prior to participation. This study has further shown how leveraging support in everyday life from one’s personal mobile device could be enhanced through a systematic approach (ie, identifying relevant support tools based on individual needs as in a typical rehabilitation process). This was exemplified by participant 3; though she already used her phone for support, the introduction of tools such as location tracking in the study considerably improved the impact this had on her quality of life.

Both familiarity and personalization were found to be important factors for acceptance in this study, echoing earlier work. These learnings offer some guidance for enhancing adoption and use in practice (eg, employing already-in-use devices, operating systems, and apps as far as possible, and adding to these only based on specific individual needs and preferences). Participant 5 showed the lowest acceptance, with several possible contributing factors, including familiarity (ie, no previous experience with smartphones) and an absence of perceived need, since she was satisfied with both her level of function and engagement (ie, following a highly active schedule) and her available support. One envisioned scenario might be that a decline in condition might motivate greater interest, though it is noted that learning to use a new tool would be increasingly challenging with worsening cognitive impairment, highlighting the importance of early adoption to enhance potential benefit.

### Future Work: Technical Development and Clinical Research

Opportunities for future work are discussed in terms of further technical development and recommended next steps for clinical research.

Two technical limitations encountered in this study are noted as key issues to address in further development:

1. Incompatibility of the monitoring system with iPhones prevented 3 participants from being able to use their own familiar device, introducing the challenge of learning to use the Android platform.
2. Unreliable data acquisition from a connected wearable limited the availability of activity data from a worn device.

A further recommendation is the development of infrastructure for feedback and sharing of behavioral data.

The early evidence presented in this study indicates feasibility of using mobile and wearable technology for support and monitoring in rehabilitation in early-stage dementia as a means toward maintaining quality of life. Recommended next steps for further research are, therefore, to conduct larger, controlled studies to provide evidence needed to confirm and quantify the impact of the described system on quality of life outcomes, such as independence, social engagement, or psychosocial symptoms of dementia such as depression. Regarding the potential for sensor-based mobility and activity monitoring to reveal patterns in behavior, this further motivates larger-scale and longer-term studies to gather the data necessary to develop algorithms for detecting and predicting changes in condition status.

### Conclusions

This work provides some of the first evidence describing the dual role of personal devices in rehabilitation for dementia by offering personalized support in everyday life and by monitoring activity and mobility behavior. Results show promise for smartphones and wearables to drive P4 approaches to dementia care. Core contributions include the following: results demonstrating that data gathered under real-life conditions are adequate for revealing behavioral patterns, initial evidence showing potential advantages of sensor-based measurement over self-reported behavior (eg, for detecting gradual trends or providing multifaceted insights into goal attainment), and qualitative reports from participants describing usefulness of the support and its impact on their health and well-being.

## Abbreviations

ACE: Addenbrooke's Cognitive Examination
CACHET: Copenhagen Centre for Health Technology
FAQ-IADL: Functional Assessment Questionnaire-Instrumental Activities of Daily Living
GPAQ: Global Physical Activity Questionnaire
GPS: global positioning system
ICT: information and communications technology
LSA: Life-Space Assessment
MCP: minimum convex polygon
MMSE: Mini-Mental State Examination
P4: predictive, preventative, personalized, and participatory
QoL-AD: Quality of Life in Alzheimer’s Disease scale
VihTek: Videncenter for velfærdsteknologi
ZBI: Zarit Burden Interview
